# My Wife and I; or Harry Henderson’s History

**Published:** 1871-01

**Authors:** Harriet Beecher Stowe

**Affiliations:** Author of “Uncle Tom’s Cabin,” “Minister’s Wooing,” &c., &c.


### MY WIFE AND I; OR HARRY HENDERSON'S HISTORY.

BY HARRIET BEECHER STOWE,

*Author of "Uncle Tom's Cabin," "Minister's Wooing," &c., &c.*

### CHAPTER I.

### INTRODUCTION.

It appears to me that the world is returning to its second childhood, and running mad for stories Stories! Stories! Stories! everywhere; stories in every paper, in every crevice, crack, and corner of the house. Stories fall from the pen faster than leaves of autumn, and of as many shades and colorings. Stories blow over here in whirlwinds from England. Stories are translated from the French, from the Danish, from the Swedish, from the German, from the Russian. There are serial stories for adults in the *Atlantic*, in the *Overland*, in the *Galaxy*, in *Harper's*, in *Scribner's*. There are serial stories for youthful pilgrims in *Our Young Folks*, the *Little Corporal*, the *Riverside*, the *Youth's Companion*, and very soon we anticipate newspapers with serial stories for the nursery. We shall have those charmingly illustrated magazines, the *Cradle*, the *Rocking-chair*, the *First Rattle*, and the *First Tooth*, with successive chapters of "Goosy Goosy Gander," and "Hickory Dickory Dock," and "Old Mother Hubbard," extending through twelve, or twenty-four, or forty-eight numbers.

I have often questioned what Solomon would have said if he had lived in our day. The poor man, it appears, was somewhat *blase* with the abundance of literature in his times, and remarked that much study was a weariness to the flesh. Then, printing was not invented, and "books" were all copied by hand, in those very square Hebrew letters, where each letter is about as careful a bit of work as a gravestone. And yet even with all these restrictions and circumscriptions, Soloman rather testily remarked, "Of making many books there is no end!" What would he have said had he looked over a modern publisher's catalogue?

It is understood now that no paper is complete without its serial story, and the spinning of these stories keeps thousands of wheels and spindles in motion. It is now understood that whoever wishes to gain the public ear, and to propound a new theory, must do it in a serial story. Hath any one in our day, as in St. Paul's, a psalm, a doctrine, a tongue, a revelation, an interpretation — forthwith he wraps it up in a serial story, and presents it to the public. We have prison discipline, free-trade, labor and capital, woman's rights, the temperance question, in serial stories. We have Romanism and Protestantism, High Church and Low Church, and no Church, contending with each other in serial stories, where each side converts the other, according to the faith of the narrator.

We see that this thing is to go on. Soon it will be necessary that every leading clergyman should embody his theology in a serial story, to be delivered from the pulpit Sunday after Sunday. We look forward to announcements in our city papers, such as these: The Rev. Dr. Ignatius, of the Church of St. Mary the Virgin, will begin a serial romance, to be entitled "St. Sebastian and the Arrows," in which he will embody the duties, the trials, and the temptations of the young Christians of our day. The Rev. Dr. Boanerges, of Plymouth Rock Church, will begin a serial story, entitled "Calvin's Daughter," in which he will discuss the distinctive features of Protestant theology. The Rev. Dr. Cool Zephyr will go on with his interesting romance of "Christianity a Dissolving View," — designed to show how everything is, in many respects, like everything else, and all things lead somewhere, and everything will finally end somehow, and that therefore it is important that everybody should cultivate general sweetness, and have the very best time possible in this world.

By the time that all these romances get to going, the system of teaching by parables, and opening one's mouth in dark sayings, will be fully elaborated. *Pilgrim's Progress* will be nowhere. The way to the celestial city will be as plain in everybody's mind as the way up Broadway — and so much more interesting! Finally, all science and all art and all business will be explained, conducted, and directed by serial stories, till the present life and the life to come shall form only one grand romance. This will be about the time of the Millennium.

Meanwhile, I am going to furnish a serial story for the *Christian Union*, and I choose the subject that is in everybody's mind and mouth, discussed on every platform, surging from everybody's tongue, and coming home to every man's business and bosom, to wit,

### MY WIFE AND I.

I trust that Miss Anthony and Mrs. Stanton, and all the prophetesses of our day, will remark the humility and propriety of my title. It is not I and My Wife — oh no! It is My Wife and I. What am I, and what is my father's house, that I should go before my wife in anything?

"But why specially for the *Christian Union?*" says Mr. Chadband. Let us in a spirit of love inquire.

Is it not evident why, O beloved? Is not that firm in human nature which stands under the title of MY WIFE AND I, the oldest and most venerable form of Christian union on record? Where, I ask, will you find a better one? — a wiser, a stronger, a sweeter, a more universally popular and agreeable one?

To be sure, there have been times and seasons when this ancient and respectable firm has been attacked as a piece of old fogyism, and various substitutes for it proposed. It has been said that "MY WIFE AND I" denoted a selfish, close corporation inconsistent with a general, all-sided, diffusive, universal benevolence; that MY WIFE AND I, in a millennial community, had no particular rights in each other more than any of the thousands of the brethren and sisters of the human race. They have said, too, that MY WIFE AND I, instead of an indissoluble unity, were only temporary partners, engaged on time, with the liberty of giving their month's notice, and starting off to a new firm.

It is not thus we understand the matter.

MY WIFE AND I, as we understand it, is the sign and symbol of more than an earthly partnership or union — of something sacred as religion, indissoluble as the soul, endless as eternity — the symbol chosen by Almighty Love to represent his redeeming, eternal union with the soul of man.

A fountain of eternal youth gushes near the hearth of every household. Each man and woman that have loved truly, have had their romance in life — their poetry in existence.

So I, in giving my history, disclaim all other. Look not for trap-doors, or haunted houses, or deadly conspiracies, or murders, or concealed crimes, in this history, for you will not find one. You shall have simply and only the old story — old as the first of Genesis, — of Adam stupid, desolate, and lonely without Eve, and how he sought and how he found her, and how they fared together thereafter.

This much, on mature consideration, I hold to be about the sum and substance of all the romances that have ever been written, and so long as there are new Adams and new Eves in each coming generation, it will not want for sympathetic listeners.

So I, Henry Henderson, — a plain Yankee boy from the mountains of New Hampshire, and at present a citizen of New York, — commence my story.

My experiences have three stages.

First, my child-wife, or the experiences of Boyhood.

Second, my shadow-wife, or the experiences of my Youth.

Third, my real wife, where I saw her, how I sought and found her, and how we fared together.

In the course of these experiences, my good friends, you will find that we take occasion to discuss all sorts of modern and exciting topics, and to keep up with the spirit of this discussing age, when there is nothing which may not be considered an open question.

The above is the introductory chapter of a new and most charming tale by Mrs. Stowe, just commenced in the *Christian Union*, and to be continued during the year 1871. For sale by all news-dealers. The publishers, Messrs. J. B. Ford & Co., 39 Park Row, New York, offer to send the paper to subscribers two months FREE; that is, all subscriptions sent in between now and January, 1871, shall be credited from the beginning of this story fully up to January, 1872. The price of an annual subscription is three dollars. Following this is the prospectus of the paper. To every new subscriber is presented *Marshall's superb "Household Engraving of Washington*," a work which has made the artist famous over Europe and America, and has never been sold for less than $5. With such a combination of attractions, it is not surprising that the *Christian Union* is taking the very front rank among religious newspapers.

